# Excess Cardiovascular Mortality in Latvia: A Novel Approach Based on Patient-Level Data to Estimate the Separate Contributions of Primary Prevention, Accessibility and Quality of Hospital Care

**DOI:** 10.34172/ijhpm.2020.229

**Published:** 2020-11-23

**Authors:** Jacopo Lenzi, Chiara Reno, Jolanta Skrule, Jana Lepiksone, Ģirts Briģis, Alina Dūdele, Maria Pia Fantini

**Affiliations:** ^1^Department of Biomedical and Neuromotor Sciences, Alma Mater Studiorum – University of Bologna, Bologna, Italy; ^2^Unit of Data Analysis of NCD and Surveys, Centre for Disease Prevention and Control of Latvia, Riga, Latvia.; ^3^Research and Health Statistics Department, Centre for Disease Prevention and Control of Latvia, Riga, Latvia.; ^4^Department of Public Health and Epidemiology, Riga Stradiņš University, Riga, Latvia.; ^5^Health Management Section, Riga Stradiņš University, Riga, Latvia.

**Keywords:** Excess Mortality, Cardiovascular Diseases, Primary Prevention, Health Services Accessibility, Quality of Care, Latvia

## Abstract

**Background:** Because quantifying the relative contributions of prevention and medical care to the decline in cardiovascular mortality is controversial, at present mortality indicators use a fifty-fifty allocation to fraction avoidable cardiovascular deaths as being partly preventable and partly amenable. The aim of this study was to develop a dynamic approach to estimate the contributions of preventable versus amenable mortality, and to estimate the proportion of amenable mortality due to non-utilisation of care versus suboptimal quality of care.

**Methods: **We calculated the contribution of primary prevention, healthcare utilisation and healthcare quality in Latvia by using Emilia-Romagna (ER) (Italy) as the best performer reference standard. In particular, we considered preventable mortality as the number of cardiovascular deaths that could be avoided if Latvia had the same incidence as ER, and then apportioned non-preventable mortality into the two components of non-utilisation versus suboptimal quality of hospital care based on the presence of hospital admissions in the days before death. This calculation was possible thanks to the availability of the unique patient identifier in the administrative databases of Latvia and ER.

**Results: **41.5 people per 100 000 population died in Latvia in 2016 from cardiovascular causes amenable to healthcare; about half of these (21.4 per 100 000) had had no contact with acute care settings, while the other half (20.1 per 100 000) had accessed the hospital but received suboptimal-quality healthcare. Another estimated 26.8 deaths per 100 000 population were due to lack of primary prevention. Deaths attributable to suboptimal quality or non-utilisation of hospital care constituted 60.7% of all avoidable cardiovascular mortality.

**Conclusion:** If research is undertaken to understand the reasons for differences between territories and their possible relevance to lower performing countries, the dynamic assessment of country-specific contributions to avoidable mortality has considerable potential to stimulate cross-national learning and continuous improvement in population health outcomes.

## Background

**Key Messages**

** Implications for policy makers**

- At present, avoidable mortality indicators use a fifty-fifty allocation to fraction premature deaths from cardiovascular causes as being partly preventable and partly amenable to healthcare.
- We show a novel approach to estimate the separate contributions of preventable versus amenable mortality, and to estimate the proportion of amenable mortality due to non-utilisation of care versus suboptimal quality of care.
- Knowing dynamic and country-specific contributions to avoidable mortality can help prioritise specific interventions and policies to reduce premature mortality.
- This assessment method has the potential to be extended to all countries that have a unique patient identifier in their administrative databases, with no need to exchange patient-level healthcare data between research agencies and member states or territories.

** Implications for the public**

When health indicators are suitable to capture aspects of care that are important to patients, the evidence culminates in concrete actions to improve the health of the population. Benchmarking against international data is further beneficial to ensuring continuous improvement in health outcomes. Avoidable (excess) mortality is a potentially useful tool to measure the effectiveness of public health and healthcare systems nationally and internationally. However, in order for this indicator to be relevant, a distinction should be made between deaths preventable through public health interventions and deaths amenable to medical care. Amenable deaths should be further apportioned in those due to suboptimal-quality services and those due to non-utilisation of services, because access is an important component of quality that drives health inequalities. In this research, we calculated these novel indicators in Latvia by using Emilia-Romagna (ER) (Italy) as the reference standard, showing their considerable potential to improve the health of the population.

Cardiovascular diseases (CVDs) account for the largest proportion of premature deaths due to non-communicable diseases worldwide.^1^ Given the multifactorial nature of CVDs and the advances in medical knowledge, treatment and technology, quantifying the relative contributions of prevention and medical care to the decline in mortality remains controversial.^2-7^ Different definitions of avoidable and/or amenable premature mortality in high-income countries have been proposed in the last decades,^8-10^ including the recent joint Organisation for Economic Co-operation and Development (OECD)/Eurostat list of causes of death.^11^ Because there is no strong evidence of predominance between the two components of primary prevention and treatment, all of these lists use a fifty-fifty allocation to fraction avoidable cardiovascular deaths as being partly preventable and partly amenable.

 The aim of this study was to develop a novel approach to estimate avoidable mortality from CVDs, namely acute myocardial infarction (AMI) and stroke. Because deaths from AMI and stroke can be avoided through both public health interventions and healthcare activities, we show how to estimate the separate contributions of preventable versus amenable mortality, and to estimate the proportion of amenable mortality due to non-utilisation of care versus suboptimal quality of care.

## Methods

###  Overview

 This retrospective study was inspired by an analysis of the *Lancet Global Health* Commission on High Quality Health Systems (HQSS) in the Sustainable Development Goal era, whose aim was to estimate deaths due to non-utilisation of healthcare and deaths due to poor quality of care in 137 low- and middle-income countries pursuing universal health coverage.^12^ To obtain these figures, the authors excluded preventable deaths by comparing mortality in each country with corresponding mortality from a reference group of 23 high-income countries with strong universal health coverage and good health outcomes.

 In this study, we adopted a similar approach to calculate the separate contributions of primary prevention, healthcare utilisation and healthcare quality in Latvia, a country in the Baltic region of Northern Europe with a population of 1.9 million. We used Emilia-Romagna (ER), a region in the northeast of Italy with 4.5 million inhabitants, as the reference standard to estimate preventable and amenable mortality in Latvia. Italy is one of the 23 countries included in the HQSS reference group, and ER is one of the highest performing among the 20 Italian regions in terms of amenable mortality and other population health profiling indicators.^13,14^ A description of the healthcare systems of Latvia and ER is provided in the next subsection.

 The main methodological difference with the HQSS is that we used patient-level data to estimate the number of incident cases and to partition the separate contributions of non-utilisation and suboptimal quality of care. This calculation was possible thanks to the availability of the unique patient identifier in the administrative databases of Latvia and ER. Contrary to the HQSS, we decided to use the expression “suboptimal quality” instead of “poor quality,” because amenable mortality indicators are intended to measure the lack of best possible healthcare.

###  Healthcare Systems of Latvia and Emilia-Romagna

 Latvia has a statutory general tax-financed healthcare system with universal population coverage and mixed public and private provision of services.^15^ The central government has a strong control over this system. In particular, the Ministry of Health defines the national health policies and coordinates the overall organisation and functioning of the healthcare system, whereas the municipalities have a limited role, mainly focussed on ensuring geographic access to healthcare services to their populations, participating in health-promotion activities and organising long-term care services.

 With the second lowest health expenditure per capita in the European Union (EU), a very low proportion of gross domestic product is spent on health (6%) and only 57% of health expenditure comes from public funding sources; as a result, Latvia’s healthcare system is critically underfunded. The public benefit package is limited, and access to inpatient and outpatient services is restricted by the annual volume quotas, thus potentially leading to long waiting times. Put together, these factors explain the high reliance on out-of-pocket spending, which represents one of the major barriers to access to healthcare services. A new price regulation amendment is going to be introduced in 2020 to improve financial access to medicines, which is an essential driver of out-of-pocket expenses.

 Despite the increased proportion of spending on outpatient care, the healthcare system of Latvia has a hospital bed capacity and utilisation persistently above the EU average. The shortages of healthcare workers are also a major concern, and the high concentration of health professionals in urban areas raises issues regarding equity and accessibility. However, the authorities have recently taken action to attract medical practitioners in rural areas.^16^

 As of 2015, Latvia had one of the highest mortality rate in the EU (1489 deaths per 100 000 population), with CVDs and cancers accounting for 59% and 20% of all deaths, respectively.^17^

 The Italian healthcare system is a national health service mainly financed through general taxation that guarantees a universal coverage to all residents. Each region is responsible for the organisation and provision of the health services included in the benefit package established by the central government (the “essential levels of care”), finances additional services and decides its own levels of co-payment for pharmaceuticals. As a result, the system is highly decentralised and gives rise to differences among regions for access to high-quality health services and for performance.

 ER is a region of Italy with a strong system of public, territorial and community welfare that faces a rapid expansion of population ageing and an increasing prevalence of multimorbidity and frailty.^18,19^ According to a 2019 national evaluation,^20^ ER is one of the three leading regions of Italy for healthcare quality, effectiveness and sustainability. It also ranks first for ability to respond to the health needs of the population and for health status maintenance.^21^

###  Definitions

 Preventable deaths are deaths that can be avoided through effective public health and primary care interventions (ie, before the onset of diseases). Because greater prevention reflects a reduction in incidence, we considered preventable mortality as the number of deaths that could be avoided if Latvia had the same incidence as the reference standard, namely ER. This approach accounts for mortality that can be prevented through public health or other upstream interventions (eg, smoking cessation, physical activity, diet, and reforms in access to pharmaceuticals), as well as for mortality that can be prevented through primary care (eg, proper hypertension management and strategies to reduce cholesterol levels). This means that preventable mortality is attributable to interventions both inside and outside the health system.

 Amenable deaths are deaths that can be avoided through timely access to good-quality healthcare. In this work, we subdivided amenable deaths into deaths due to non-utilisation of services and deaths due to use of suboptimal-quality services. More specifically, we estimated avoidable deaths that were amenable to hospital care in terms of accessibility to and quality of available services.

 Causes of death preventable and/or amenable to healthcare include, among the others, diseases of the circulatory system, cancers, infectious diseases, endocrine/metabolic diseases, and diseases of the digestive system. We focussed our analyses on AMI and stroke because knowing incidence is crucial to partition the separate components of avoidable (excess) mortality. In fact, while incidence of acute care conditions is relatively easy to estimate from administrative databases, other conditions such as cancers require the implementation of dedicated registers. CVDs, however, are responsible for the largest portion of amenable deaths—33% in low- and middle-income countries and 50% in EU countries.^12,17^

 For simplicity’s sake, the statistical methods used to estimate AMI mortality and stroke mortality are presented in two separate subsections.

###  Statistical Analysis: Acute Myocardial Infarction Mortality

 In keeping with the Global Burden of Disease (GBD) study,^22^ incident cases were defined as the sum of admissions to hospital with a primary diagnosis of AMI (from hospital administrative databases) and deaths from AMI (from cause of death register) in the year 2016. Incident cases had no hospital admissions for AMI over the previous 28-day period, which corresponds to the acute and subacute phases of the disease.^22^ Simply put, there had to be a timespan of >28 days between AMI cases for the same patient. The codes for identification of AMI were 410 (ICD-9) or I21-I22 (ICD-10).

 Cases were excluded if any of the following criteria were met:

- Age >74 years, ie, the age threshold for premature versus non-premature deaths
- Scheduled hospital admission, ie, non-incident case of AMI
- Length of hospital stay <2 days and discharged home, ie, potential erroneous diagnosis
- Non-resident in the country
- Missing information on sex.

Preventable deaths were defined as 28-day AMI deaths among preventable cases. Preventable cases are function of the excess incidence in Latvia (*LV*) compared to the incidence in ER (*ref*). Simply put, preventable cases are those that could be avoided if Latvia had the same incidence as ER. The formula to get the number of preventable cases was defined as follows:


$$Cases_{ij}^{LV,prev}=\left(1-\frac{I_{ij}^{ref}}{I_{ij}^{LV}}\right)\times Cases_{ij}^{LV}$$

for age group *i* and sex *j*, where Cases is the number of incident cases and *I* is the incidence. If $I_{ij}^{ref}>I_{ij}^{LV},Cases_{ij}^{LV,prev}$ was set to 0.

 Preventable deaths were calculated as:


$$Deaths_{ij}^{LV,prev}=Cases_{ij}^{LV,prev}\times CF_{ij}^{LV}$$

Where *CF* is 28-day case fatality.

 Amenable deaths were defined as 28-day AMI deaths among non-preventable cases, that is, the ones that remained after excluding preventable cases:


$$Cases_{ij}^{LV,amen}=Cases_{ij}^{LV}-Cases_{ij}^{LV,prev}$$

Assuming that non-preventable cases have *CF* equivalent to that of preventable cases (since they share the same healthcare and social context), amenable deaths were calculated as:


$$Deaths_{ij}^{LV,amen}=Cases_{ij}^{LV,amen}\times CF_{ij}^{LV}$$

Amenable deaths were then apportioned into two components: deaths due to non-utilisation of hospital services (*Deaths*^amen(NU)^) and deaths due to suboptimal-quality hospital services (*Deaths*^amen(SQ)^). The formulae to get these estimates were:


$$Deaths_{ij}^{LV,amen\left(NU\right)}=Cases_{ij}^{LV,amen}\times \frac{Deaths_{ij}^{LV,w/o}}{Cases_{ij}^{LV}}$$

$$Deaths_{ij}^{LV,amen\left(SQ\right)}=Cases_{ij}^{LV,amen}\times \frac{Deaths_{ij}^{LV,w/o}}{Cases_{ij}^{LV}}$$

Where *Deaths*^w/o^ is the number of deaths with no hospital admissions for AMI over the previous 28-day period (ie, patients who did not make it to the hospital), and *Deaths*^w/o^ is the number of deaths with hospital admissions for AMI over the previous 28-day period (ie, patients who made it to the hospital but died within 28 days of admission). Instead of using individual data, the HQSS used aggregate data such as global estimates and household population surveys to gather information about healthcare utilisation.^12^ Our approach might overestimate the impact of non-utilisation of healthcare, because the first signs of the disease can be detectable during other contacts with the health services; however, adequate access to emergency services for timely initiation of appropriate treatment represents a major determinant for cardiovascular mortality.

 Mortality rates were calculated as the number of deaths per 100 000 resident population, overall and by sex. Mortality rates attributable to non-utilisation and to suboptimal quality were provided not only for Latvia, but also for ER. Confidence intervals (CIs) were estimated using the bias-corrected bootstrap method and expressing the upper and lower bounds on the logit scale.^23^

 In a sensitivity analysis, instead of quantifying amenable deaths as:


$$Deaths_{ij}^{LV,amen}=Cases_{ij}^{LV,amen}\times CF_{ij}^{LV}$$

we used this formula:


$$Deaths_{ij}^{LV,amen}=Cases_{ij}^{LV,amen}\times \left(CF_{ij}^{LV}-CF_{ij}^{ref}\right)$$

This alternative approach, which is the one used by Kruk and colleagues,^12^ computes amenable mortality as excess mortality among non-preventable cases (excess mortality is the surplus of case fatality compared to the case fatality in the reference group). The rationale behind it is the prioritisation of primary prevention, which is suitable when low- or middle-income countries are analysed.

 All data were analysed using Stata version 15 (StataCorp. 2017. *Stata Statistical Software: Release 15.* College Station, TX: StataCorp LLC). The code to obtain the number of incident cases and deaths is presented in Supplementary file 1.

###  Statistical Analysis: Stroke Mortality

 The methods used to estimate stroke mortality were identical to those used for AMI mortality. However, according to the definition proposed by the GBD,^22^ incident cases had no hospital admissions for stroke in the previous year, and consequently 1-year case fatality was investigated.

 All analyses were carried out separately for haemorrhagic stroke (ICD-9/10 430-432/I60-I62) and ischaemic stroke (ICD-9/10 433-434,436/I63-I64). Unspecified strokes (ICD-9/10 436/I64) were classified as ischaemic strokes.^24^

## Results

 We estimated that there were 1209 avoidable deaths from AMI and stroke in Latvia in 2016 (95% CI: 1141-1280), including 475 (39.3%) deaths preventable through primary prevention (95% CI: 430-515) and 734 (60.7%) deaths amenable to healthcare (95% CI: 683-789). Of the excess deaths amenable to healthcare, an estimated 379 (51.6%) were due to non-utilisation of hospital care (95% CI: 343-416), whereas 355 (48.4%) were due to suboptimal quality of available hospital care (95% CI: 322-394).

 Table shows avoidable deaths stratified by condition. Deaths from stroke made up 60.6% (n= 733) of the avoidable cardiovascular deaths in Latvia, of which 65.3% (n= 479) were from ischaemic stroke; the remaining 476 excess deaths (39.4%) were from AMI. Most excess deaths from ischaemic stroke were accounted for by lack of primary prevention (n= 249, 52.0% of all excess mortality). Non-utilisation of acute hospital care contributed to the most deaths from AMI (n= 267, 56.1%), while suboptimal-quality hospital care contributed to the most deaths from haemorrhagic stroke (n= 109, 42.9%).

**Table:** Preventable and Amenable Cardiovascular Deaths, and Deaths Related to Use of Suboptimal-Quality Hospital Services Versus Non-utilisation of Hospital Services, by Condition (Latvia, 2016)

**Cause of Death**	**Avoidable Deaths**	**Preventable Deaths**	**Amenable Deaths**		
			**Total**	**Due to Non-utilisation of Hospital Services**	**Due to Use of Suboptimal-Quality Hospital Services**
AMI	476	132 (27.7%)	344 (72.3%)	267 (77.6%)	77 (22.4%)
Haemorrhagic stroke	254^a^	94 (37.0%)	160 (63.0%)	51 (31.9%)	109 (68.1%)
Ischaemic stroke	479^a^	249 (52.0%)	230 (48.0%)	61 (26.5%)	169 (73.5%)

Abbreviation: AMI, acute myocardial infarction. ^a^ Of the 733 avoidable deaths from stroke, 665 (90.7%) occurred within 28 days of hospital admission. Of the 733 avoidable deaths from stroke, 665 (90.7%) occurred within 28 days of hospital admission.

**Figure 1:**
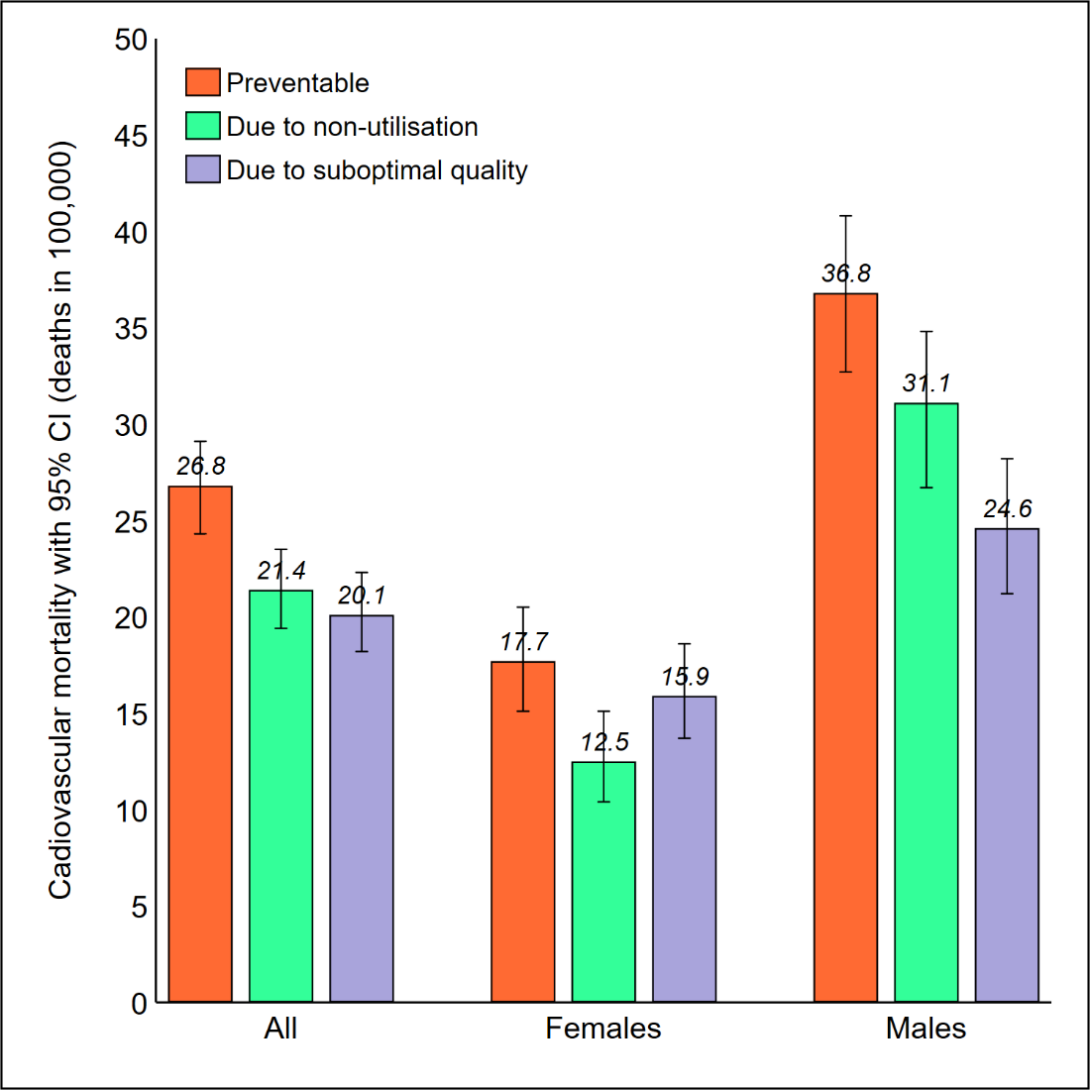
Cardiovascular Mortality Due to Lack of Primary Prevention, Non-utilisation of Hospital Care, and Access to Suboptimal-Quality Hospital Care, by Sex (Latvia, 2016).

Figure 1 illustrates the cardiovascular mortality per 100 000 population due to lack of primary prevention, non-utilisation of hospital care, and access to suboptimal-quality hospital care, by sex. The avoidable mortality rate among men was twice the avoidable mortality rate among women (92.4 versus 46.2 deaths in 100 000). The partition of amenable mortality was different in men and women: most of the women’s deaths were related to suboptimal quality (15.9 deaths in 100 000), while most of the men’s deaths were related to non-utilisation (31.1 deaths in 100 000).

 In ER, the best performer reference standard, there were 13.7 deaths in 100 000 due to non-utilisation of hospital care (95% CI 12.7-15.0) (females: 7.6; males: 19.9) and 6.7 deaths in 100 000 due to use of suboptimal-quality hospital care (95% CI 5.9-7.5) (females: 5.0; males: 8.4). By construction, no death in the reference standard was considered as preventable.

 The incidence rates of Latvia and ER, which were calculated to apportion the contributions of primary prevention and healthcare in Latvia, are presented in Supplementary file 2.

###  Sensitivity Analysis

**Figure 2:**
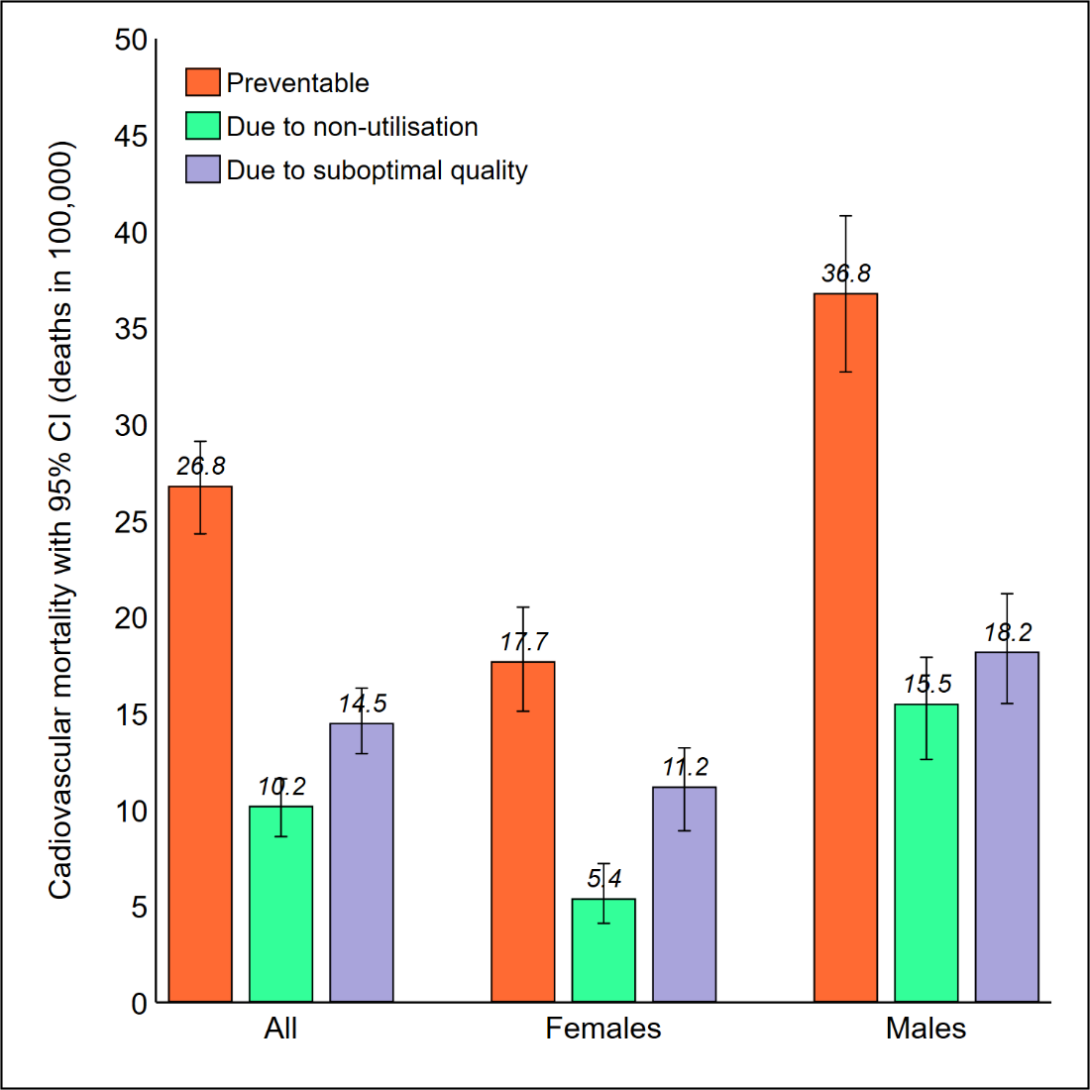
Cardiovascular Mortality Due to Lack of Primary Prevention, Non-utilisation of Hospital Care, and Access to Suboptimal-Quality Hospital Care, by Sex (Latvia, 2016); Mortality Amenable to Healthcare is the Surplus of Case Fatality Compared to the Case Fatality in the Reference Standard.

 We recalculated amenable mortality in Latvia as the surplus of case fatality among non-preventable incident cases, as compared to ER. Using this approach, the number of deaths amenable to healthcare declined from 734 to 438 (–40.3%). As illustrated in Figure 2, the overall mortality rate due to non-utilisation of hospital care was reduced by half (21.4 to 10.2 deaths in 100 000), while the mortality rate due to access to suboptimal-quality hospital care was reduced by about a quarter (20.1 to 14.5 deaths in 100 000). As a result, suboptimal quality contributed to more deaths than non-utilisation.

## Discussion

 41.5 people per 100 000 population died in Latvia in 2016 from cardiovascular causes amenable to healthcare; about half of these people (21.4 per 100 000) had had no contact with acute care settings, while the other half (20.1 per 100 000) had accessed the hospital but received suboptimal-quality healthcare. Another estimated 26.8 deaths per 100 000 population were due to lack of primary prevention. Deaths attributable to suboptimal quality or non-utilisation of hospital care constituted 60.7% of all avoidable cardiovascular mortality in the country.

 Our findings cannot be directly compared with the HQSS analysis of amenable mortality, which involved countries classified as low- or middle-income.^12^ However, Kruk and colleagues found that at global level 28 deaths per 100 000 population occurred for cardiovascular causes amenable to healthcare services, a value strikingly close to the mortality rate that we found in our sensitivity analysis (24.7 deaths in 100 000). This value was obtained by computing amenable mortality in Latvia as excess case fatality among non-preventable cases. Despite this similarity, the contribution of suboptimal-quality hospital services was much lower in Latvia (59% versus 84%), strengthening the evidence that suboptimal-quality healthcare is a lesser impediment to improved population health in higher- than in lower-income countries.

 The reason why we presented two alternative methods to estimate the number of deaths amenable to healthcare services needs clarification. The method used by the HQSS minimises amenable mortality in order to prioritise primary prevention, which is cheaper and often more effective than treatment.^12^ This should be the method of choice when there are large gaps between the healthcare system performance of the study countries and the best performing comparator(s), because a direct comparison of case fatality rates attributable to health services would be unrealistic. On the other hand, when more developed countries such as Latvia are analysed, our assessment method can provide less conservative estimates and lead to more informative conclusions about the health impact of suboptimal quality and non-utilisation.

###  Preventable Cardiovascular Mortality

 The large number of preventable deaths in Latvia might have to do with the high prevalence of cardiovascular risk factors in the country as compared to the reference standard.^16,25^ One in three persons in Latvia is a smoker as compared to one in four in ER.^26,27^ In Latvia, the prevalence of overweight in adults is 60% (30% in ER) and the prevalence of obesity is 25% (13% in ER).^27,28^ Poor nutritional habits and physical inactivity can partly explain the high percentage of overweight and obesity: the share of the population consuming fruit and vegetables every day is 60% in Latvia and virtually 100% in ER^16,27^; the prevalence of sedentary lifestyle is one in three in Latvia and one in five in ER.^26,27^ A major risk factor for stroke is hypertension, which has been reported to be present in 35% of Latvia’s population and in 28% of ER population.^16,27^ Another condition that increases the risk of stroke and also of AMI is diabetes mellitus,^29^ which is more prevalent in Latvia (9%) than in ER (7%).^27,28^

 Socioeconomic inequalities widely contribute to health risks in Latvia.^16^ The higher prevalence of behavioural risk factors among people with low education and income leads to increased mortality from CVDs and different types of cancers, resulting in a considerable gap in life expectancy between socioeconomic groups.^16,30^ In 2014, for instance, the prevalence of daily smoking and obesity among adults who had not completed secondary education was 24% and 23% respectively, compared to 14% and 18% among those with tertiary education.^16,30^ A study investigating the social determinants of health behaviours in Finland and the Baltic countries in the years 1998-2008 showed that, in Latvia, the consumption of fresh vegetables was lower in the less educated groups.^31^ Strong inequalities also persist in the prevalence of chronic conditions such as diabetes.^30^

 Because behavioural risk factors have a strong association with CVDs, they should be targeted as a public health priority to reduce the incidence of such conditions and the health inequalities they lead to. Steps have been taken in Latvia to implement awareness campaigns and strengthen primary prevention strategies. For example, the Public Health Strategy for 2014-2020 allocated resources to health promotion activities targeting tobacco consumption.^16^ It is also essential to promote patients’ education and collaboration with the general practitioners to achieve an optimal management of the conditions associated with an elevated risk of CVD. According to a recent cross-sectional study,^32^ nearly half of the patients with hypertension in Latvia were non-adherent to medications; frequently mentioned reasons for interrupting or ending the therapy were forgetfulness, concerns about getting addicted, and undesirable side effects. Moreover, not all patients passed on information to their physicians about concurrent medications or food supplements.

 Our study findings show that primary prevention had a differential impact on condition-specific premature deaths: low for AMI (27.7%) and high for ischaemic stroke (52.0%). On one hand, in ER some risk factors for AMI such as tobacco use, hypertension and diabetes, albeit lower than in Latvia, are still quite common, and some others such as hyperlipidaemia are alarmingly high.^27^ On the other hand, as already mentioned, the high incidence of ischaemic stroke in Latvia asks for specific considerations regarding the proper treatment and monitoring of hypertension and atrial fibrillation, which are two difficult conditions to manage. According to the non-governmental organisation “ParSirdi.lv,” that brings together patients with various heart and CVDs, a great issue is that, despite the availability of effective treatment for stroke risk factors, often patients cannot afford to pay for them and stop or do not even start taking medications.^33^ Furthermore, many patients do not qualify for medication reimbursement due to very strict national requirements.

###  Amenable Mortality From Acute Myocardial Infarction

 Strengthening prehospital assistance and improving nationwide distribution of specialised centres should be set as priorities to reduce the number of amenable deaths from AMI, which in Latvia were mostly due to non-utilisation of hospital care (77.6%). In 2014, there were five cath labs in three hospitals at a distance of about 200 km from each other; three of these labs were not open 24/7 and one provided only angiography services.^34^

 Rapid movement through the care pathway is essential because the myocardium has to be saved in a very short time before being irreparably damaged. Optimal emergency management of AMI can be summarised into four key elements^35,36^: early patient recognition of symptoms and rapid request for medical assistance, quick arrival of well-trained medical personnel, prompt transport to the hospital, and rapid reperfusion treatment.

###  Amenable Mortality From Stroke

 The use of suboptimal-quality services in Latvia accounted for the greatest part of amenable deaths from both haemorrhagic (68.1%) and ischaemic stroke (73.5%). This finding suggests that patients with stroke, as opposed to those with AMI, reach the hospital more often. However, in 2016 one in three patients were not taken directly to a stroke unit, and only one hospital had a 24/7 thrombectomy service.

###  Sex Differences

 The greater exposure to key risk factors among men and male sex being a risk factor itself for both AMI and stroke,^16,25^ could account for sex differences in premature cardiovascular mortality. The large differences found in amenable mortality due to non-utilisation of hospital care (females: 12.5 per 100 000; males: 31.1 per 100 000) might be explained by the fact that women seek more healthcare in response to health concerns and are more adherent to medical recommendations.^37^

###  Amenable Mortality in the Reference Standard

 We found that, in ER, 20.4 cardiovascular deaths in 100 000 were amenable to hospital care. This low figure might reflect the fact that specific health services to optimise the management of AMI and stroke have been operational for several years in the region.

 An urgent and emergency care network for cardiac services was established in 2003 to reduce “avoidable delays” and promote early revascularisation techniques, such as primary angioplasty.^38^ The organisational structure of the network is based on the hub-and-spoke model; highly complex interventions are performed in 16 centres of excellence (hubs) while peripheral units (spokes) select and possibly refer the patients to these centres.^39^ The cardiac network appeared to be effective: mortality following AMI decreased by 39% over thirteen years and nowadays two thirds of ST elevation MIs undergo primary angioplasty, while 90% of the patients are treated in cardiovascular intensive care units.^38^

 An integrated care pathway for stroke, which comprises prehospital assistance, stroke unit care, intensive rehabilitation, early supported discharge and follow-up services, was set up in 2007.^40^ There are currently 13 stroke units (hubs) delivering thrombolysis, of which five can also perform thrombectomy. To date, 79% of patients are treated in a stroke unit and 16% receive intravenous thrombolytic therapy.^40^

###  Strengths and Limitations

 The results of this study should be interpreted in light of its strengths and limitations. Instead of adopting a fifty-fifty allocation to fraction deaths as being partly preventable and partly amenable, we used patient-level data to calculate preventable mortality as function of excess incidence compared to a reference standard. Another strength of our analysis is that mortality for users versus non-users was assessed by checking for the presence of hospital admissions for specified conditions in the days before death.

 This analysis was made possible by the presence of a unique patient identifier in the administrative databases of Latvia and ER. The use of data linkage is both a strength and a limitation, because to date a large number of countries do not have access to the routine use of a unique patient identifier, especially those that would benefit more from the implementation of healthcare quality and access measures. The most reliable estimates based on aggregate data are those of the HQSS, which integrated data from the GBD, the World Health Survey and other secondary sources to produce a comprehensive list of health-system profiles for low- and middle-income countries.^12,41^ Coding quality is another issue when using administrative data — according to Bārzdiņš and colleagues,^42^ under-reporting of non-ST elevation myocardial infarctions in Latvia might lead to overestimation of 30-day mortality rates in public report cards. However, international comparisons stimulate constant revisions of coding accuracy to improve the robustness of health indicators derived from administrative data.^43^ An important limitation is that we analysed condition-specific deaths, and even in the presence of strong vital registration systems, records can misidentify the underlying cause of death and lead to some bias if comorbidities and disease history are different between lower performing countries and the reference set. Another limitation of this method is that it is not intended to disentangle the separate contributions of public health, inter-sectoral upstream policies and primary care to preventable mortality, unless some policy-meaningful reference groups of countries are used for comparison.

 With regard to the reference countries, a potential limitation of our analysis is the use of the Italian region of ER, whose choice was based more on convenience than on performance-oriented considerations. Were this technique to be extended on a large scale, a policy-meaningful reference group should be made up of top-ranking countries or territories in terms of health status, health expenditure, risk factors for health, quality and outcomes of care, access to care, and healthcare activities. To reduce the possible effect of non-modifiable risk factors of disease, such as genetics, the eligible countries should be chosen to be geographically distant.

 This study cannot be seen as a full and systematic analysis of excess premature mortality in a country, because AMI and stroke, albeit highly prevalent, are only two of the causes of death that are avoidable in people <75 years.^8,11,44-47^ Evaluating other diseases would require the integration of different data sources, such as cancer registers for cervical cancer or prescription databases and primary-care databases for diabetes mellitus.

 Because AMI and stroke arise acutely and require time-dependent acute care, we designed amenable mortality as a measure of access to and quality of hospital care. This approach can overestimate the impact of non-utilisation of healthcare on mortality, because the first signs of the disease might be detectable during other contacts with the health services, such as outpatient office visits or hospitalisations for different conditions. However, because prompt treatment is a cornerstone of the management of AMI and stroke, calling the emergency ambulance service in response to symptoms should always be the first course of action to reduce prehospital delay times. Another potential limitation in our study is that we analysed 1-year stroke mortality, which is not a suitable indicator of hospital care quality. However, we found that the vast majority of deaths (>90%) occurred within a few weeks of hospital admission.

 Some other limitations of these metrics are common to other mortality indicators used to assess the performance of healthcare systems. First, not all deaths from potentially avoidable causes can actually be avoided; for instance, some deaths may be untreatable due to concurrent health problems, while some deaths could be due to events against which no protective measure could have been taken. This leads to an overestimation of all the components of avoidable mortality. Second, the mutually exclusive nature of these components does not imply that all cases assigned to the preventable group do not have an amenable component, and vice versa. Third, the choice of a reference age to determine premature deaths is necessary but simplistic, because deaths over this age can be premature if the health status of the deceased was good. Moreover, a lower cut-off (eg, 70 years in place of 75) might be considered when low-income countries and deprived areas are analysed.

## Conclusion

 It is possible to estimate the contributions of primary prevention, accessibility and quality of hospital care to premature cardiovascular mortality using real-world data, although some major limitations need to be borne in mind. When applied to Latvia’s healthcare system performance assessment, this index has been shown to be helpful for setting health priorities and identifying entry points for health-system improvement.

 A dynamic assessment of country-specific contributions to avoidable mortality would stimulate cross-national learning and strengthen international benchmarking of performance, with no need to exchange patient-level healthcare data between research agencies and member states or territories.

## Acknowledgements

 The authors would like to thank Ian Brownwood of the OECD, for helpful discussion and criticism.

## Ethical issues

 Access to Latvia’s administrative data was regulated by the European Commission’s health systems performance assessment project “Developing Health System Performance Assessment for Slovenia and Latvia” (grant agreement: SRSS/S2017/019). Access to ER administrative data was conducted in conformity with the Italian Privacy Code (Legislative decree 196/2003, amended by Legislative Decree 101/2018), which exempts from the obligation to seek written informed consent and approval from the ethics committee when using pseudonymised data that are primarily collected for healthcare management and healthcare quality evaluation and improvement. According to Articles 99–110-bis on medical, biomedical and epidemiological research (Legislative Decree 101/2018), when investigators use data collected by healthcare systems or previous studies, consulting all the participants would represent a disproportionate effort, considering that safeguards such as key-coding (pseudonymisation) are in place to protect the data.

## Competing interests

 Authors declare that they have no competing interests.

## Authors’ contributions

 J Lenzi developed the concept for this study, conducted data analysis, contributed to the interpretation of the results, and wrote the first draft. CR and MPF contributed to the interpretation of the results, drafted sections of the paper and revised it critically for important intellectual content. JS and J Lepiksone contributed to the acquisition of data and revised the paper critically for important intellectual content. ĢB and AD contributed to the interpretation of the results and revised the paper critically for important intellectual content. All authors approved the final version for submission.

## Disclaimer

 The views expressed in the submitted article are the authors’ and not an official position of their respective institutions.

## Authors’ affiliations

^1 ^Department of Biomedical and Neuromotor Sciences, Alma Mater Studiorum – University of Bologna, Bologna, Italy. ^2^Unit of Data Analysis of NCD and Surveys, Centre for Disease Prevention and Control of Latvia, Riga, Latvia. ^3^Research and Health Statistics Department, Centre for Disease Prevention and Control of Latvia, Riga, Latvia. ^4^Department of Public Health and Epidemiology, Riga Stradiņš University, Riga, Latvia. ^5^Health Management Section, Riga Stradiņš University, Riga, Latvia.

## Supplementary files

Supplementary file 1. Stata Code to Estimate the Number of Incident Cases and Premature Deaths from Acute Myocardial Infarction in Latvia in 2016, by Age, Sex and Presence/Absence of Hospital Admissions in the Previous 28-Day Period.

Supplementary file 2. Incidence Rates (Per 100 000 Inhabitants) of Cardiovascular Diseases in Latvia and Emilia-Romagna (Year 2016), by Sex.
