# Supplementary material for: Excess Cardiovascular Mortality in Latvia: A Novel Approach Based on Patient-Level Data to Estimate the Separate Contributions of Primary Prevention, Accessibility and Quality of Hospital Care
Source: Int J Health Policy Manag. 2020 Nov 23;11(6):820–8. doi: 10.34172/ijhpm.2020.229 (PMC9309914; doi:10.34172/ijhpm.2020.229)
Supplement: Supplementary file 1 — Stata Code to Estimate the Number of Incident Cases and Premature Deaths from Acute Myocardial Infarction in Latvia in 2016, by Age, Sex and Presence/Absence of Hospital Admissions in the Previous 28-Day Period. [file ijhpm-11-820-s001.pdf]

Supplementary file 1. Stata Code to Estimate the Number of Incident Cases and Premature Deaths from Acute Myocardial Infarction in Latvia in 2016, by Age, Sex and Presence/Absence of Hospital Admissions in the Previous 28-Day Period

```
/*
To run this code, two sets of data are necessary:
1) ami_hosp_lv_2016: hospital admissions for AMI between 1/12/15 and 31/12/16
   (source: Latvia's hospital discharge abstracts)
2) ami_death_lv_2016: deaths from AMI between 1/1/16 and 31/1/17 (source:
   Latvia's vital registration system)
Exclusion criteria 1-5 are already applied (see the Statistical Analysis subsection)
*/

/*
Set (1) includes the following:
+ id: unique patient identifier
+ sex: patient sex
+ age_group: patient age in classes
+ dt_adm: admission date
*/

/*
Set (2) includes the following:
+ id: unique patient identifier
+ sex: patient sex
+ age_group: patient age in classes
+ dt_death: death date
*/

* Data preparation
use ami_hosp_lv_2016, clear          // load hospital data
generate dt_event = dt_adm           // duplicate admission date as dt_event
generate data = 1                    // useful for upcoming sorting
save ami_hosp_lv_2016, replace       // overwrite data
use ami_death_lv_2016, clear         // load mortality data
generate dt_event = dt_death         // duplicate death date as dt_event
generate data = 2                    // useful for upcoming sorting
save ami_death_lv_2016, replace      // overwrite data
use ami_hosp_lv_2016, clear          // reload hospital data
append using ami_death_lv_2016       // append mortality data
sort id dt_event data                // arrange by UPI, date and data source
bysort id: generate J = _N           // number of records for each UPI
bysort id: generate j = _n           // record number for each UPI (oldest to newest)
generate incident = 1                // tag all records as incident cases
replace incident = 0 if             /// turn into non-incident if...
id == id[_n-1] &                    /// ... there is another AMI event...
(dt_event - dt_event[_n-1]) <= 28    // ... in the previous 28-day period
replace incident = . if             /// incident status is missing if...
year(dt_event) != 2016               // ... the event occurred in 2015 or 2017
rename dt_event dt_ami               // change name for upcoming merging
rename dt_death dt_death1            // change name for upcoming merging
merge m:1 id using ami_death_lv_2016 // many-to-one merge with mortality data
generate death28days = 0
replace death28days = 1             ///
if dt_death - dt_ami <= 28           // death within 28 days of AMI date
generate hosp = 1
replace hosp = 2 if dt_death1 != .   // presence/absence of hospital admissions...
label define hosp 1 "w/" 2 "w/o"     // ... for AMI in the previous 28-day period
label values hosp hosp
save ami_lv_2016                     // save new dataset

* Results
tab age_group sex if incident == 1   // incident cases by age and sex
table age_group sex hosp if         ///
incident == 1 & death28days == 1, m  // deaths by age, sex and hosp (w/ or w/o)

/*
Please note: if you suspect inconsistencies in age and sex reported on hospital and mortality
data, use different names (e.g., sex1 for hospital data and sex2 for mortality data) and
cross-tabulate variables for data quality control
*/
```

```
/*
Please also note that the following adjustments are needed to analyse stroke data:
+ hospital data include all admissions between 1/1/15 and 31/12/16
+ mortality data include all deaths between 1/1/16 and 31/12/17
+ cases are non-incident if there are other stroke events in the previous 365 days
*/
```
