# Supplementary material for: Excess Cardiovascular Mortality in Latvia: A Novel Approach Based on Patient-Level Data to Estimate the Separate Contributions of Primary Prevention, Accessibility and Quality of Hospital Care
Source: Int J Health Policy Manag. 2020 Nov 23;11(6):820–8. doi: 10.34172/ijhpm.2020.229 (PMC9309914; doi:10.34172/ijhpm.2020.229)
Supplement: Supplementary file 2 — Incidence Rates (Per 100 000 Inhabitants) of Cardiovascular Diseases in Latvia and Emilia-Romagna (Year 2016), by Sex. [file ijhpm-11-820-s002.pdf]

Supplementary file 2. Incidence Rates (Per 100 000 Inhabitants) of Cardiovascular Diseases in Latvia and Emilia-Romagna (Year 2016), by Sex

Disease	Latvia			Emilia-Romagna		
	All	Females	Males	All	Females	Males
Acute myocardial infarction	133.3[a]	63.2	209.7	116.5[b]	52.8	180.8
Haemorrhagic stroke	36.8	31.7	42.4	27.4	22.9	31.9
Ischaemic stroke	148.2	114.8	184.6	83.0	59.0	107.3

[a] 5.1% of the incidence was made up of recurrent events for the same patient over the year.
[b] 2.3% of the incidence was made up of recurrent events for the same patient over the year.
